# Use of Self-Care and Practitioner-Based Forms of Complementary and Alternative Medicine before and after a Diagnosis of Breast Cancer

**DOI:** 10.1155/2013/301549

**Published:** 2013-08-12

**Authors:** Alissa R. Link, Marilie D. Gammon, Judith S. Jacobson, Page Abrahamson, Patrick T. Bradshaw, Mary Beth Terry, Susan Teitelbaum, Alfred Neugut, Heather Greenlee

**Affiliations:** ^1^Department of Epidemiology, Mailman School of Public Health, Columbia University, 722 West 168th Street, New York, NY 10032, USA; ^2^Department of Epidemiology, University of North Carolina, 2101 McGavran-Greenberg Hall, CB No. 7435 Chapel Hill, NC 27599, USA; ^3^Department of Nutrition, Department of Epidemiology, University of North Carolina, 2200 McGavran-Greenberg Hall, CB No. 7461 Chapel Hill, NC 27599, USA; ^4^Department of Preventive Medicine, Mount Sinai School of Medicine, 17 E 102nd Street, New York, NY 10029, USA; ^5^Department of Medicine, College of Physicians and Surgeons, Columbia University, 722 West 168th Street, New York, NY 10032, USA

## Abstract

*Purpose*. We examine factors associated with self-care, use of practitioner-based complementary and alternative medicine (CAM), and their timing in a cohort of women with breast cancer. *Methods*. Study participants were women with breast cancer who participated in the Long Island Breast Cancer Study Project. Self-care is defined as the use of multivitamins, single vitamins, botanicals, other dietary supplements, mind-body practices, special diets, support groups, and prayer. Within each modality, study participants were categorized as continuous users (before and after diagnosis), starters (only after diagnosis), quitters (only before diagnosis), or never users. Multivariable logistic regression was used for the main analyses. *Results*. Of 764 women who provided complete data, 513 (67.2%) initiated a new form of self-care following breast cancer diagnosis. The most popular modalities were those that are ingestible, and they were commonly used in combination. The strongest predictor of continuous use of one type of self-care was continuous use of other types of self-care. Healthy behaviors, including high fruit/vegetable intake and exercise, were more strongly associated with continuously using self-care than starting self-care after diagnosis. *Conclusions*. Breast cancer diagnosis was associated with subsequent behavioral changes, and the majority of women undertook new forms of self-care after diagnosis. Few women discontinued use of modalities they used prior to diagnosis.

## 1. Introduction

Over the past two decades, the use of complementary and alternative medicine (CAM) practices—modalities used for health and wellbeing that are considered outside the realm of conventional medicine—has been steadily rising in the United States [1–3]. Patients with cancer and other chronic diseases are more likely to use CAM than are those without chronic illness [4], and breast cancer patients are more likely to use CAM than patients with colon [5], prostate [5], or gynecological [6] cancers. Estimates of CAM use among women with breast cancer range from 48% to 86% [7–9]. 

The frequency of CAM use among breast cancer patients is not surprising in the light of the physical and emotional burden that breast cancer entails. Breast cancer patients report using CAM for recovery, healing, improving health, strengthening the immune system, reducing side effects of cancer treatments, reducing physical and psychological distress [10], and increasing feelings of control [10, 11]. CAM encompasses a vast array of modalities, some of which require a CAM practitioner or provider, a commitment of patient and practitioner time, and practitioner payment; only some states in the US mandate health insurance coverage of CAM practitioners. Other modalities fall into the category of self-care, a range of activities in which individuals engage autonomously and regularly to maintain or improve health, beauty, spiritual connection, or general wellbeing. 

Due to the fact that large numbers of cancer patients use CAM, it is increasingly important to develop epidemiological methods to both describe and analyze CAM use patterns. Historically, most analyses of CAM use among cancer populations have focused on use after diagnosis. However, it is important to consider postdiagnosis CAM use in context of the patient's behavior prior to a cancer diagnosis. In this paper, we present a novel approach to describe changes in CAM use before and after breast cancer diagnosis. Furthermore, since CAM encompasses a wide variety of individual modalities that have different implications on pathophysiology and treatment course, we propose that it is important to consider changes in CAM use across specific categories of CAM. 

Using follow-up data from the Long Island Breast Cancer Study Project, we analyzed predictors of self-care practices and practitioner-based modalities of CAM before and after a first breast cancer diagnosis. Most studies of CAM use do not separate self-care (i.e., modalities that patients can access and use on their own) from practitioner-based modalities. Our primary aim was to analyze the determinants of self-care versus practitioner-based modalities; other aims were to determine how use of these modalities changed after the breast cancer diagnosis and how use of self-care and practitioner-based modalities might be associated with health-related behaviors such as exercise, smoking, and alcohol intake. 

## 2. Methods

### 2.1. Participants

The Long Island Breast Cancer Study Project (LIBCSP) originated as a population-based, case-control study of women residing in Nassau or Suffolk county, New York, USA [12]. The LIBCSP was federally mandated to investigate the possibility of associations between environmental toxicants and the high incidence of breast cancer on Long Island. Eligible cases were women diagnosed with first primary in situ or invasive breast cancer between August 1996 and July 1997. Potential cases were identified through frequent communication with pathology departments at every hospital on Long Island, and 3 major tertiary care facilities in New York City. The physicians of potentially eligible women confirmed diagnoses and gave permission to contact eligible cases; 82.1% of eligible subjects with a consenting physician agreed to participate in the study. The study enrolled 1508 cases [12].

Between 2002 and 2004, a follow-up study was conducted among the LIBCSP cases [13]. Of the 1414 cases (93.8%) who agreed to future contact, 1098 (77.4%) participated in the follow-up, but 334 (23.6%) completed only a short-form interview or had a proxy interview (completed by a relative on their behalf). Only those who completed the full follow-up telephone interview [14] were included in the analysis reported here. 

Institutional review boards at all participating institutions approved the study protocol, and all participants provided written informed consent prior to the in-person baseline interview and verbal consent prior to the telephone follow-up interview. 

### 2.2. Data Collection

#### 2.2.1. Baseline Questionnaire

The baseline questionnaire was administered to cases by a trained interviewer in the respondent's home soon after a first primary breast cancer diagnosis (mean = 89 days among the subsample with a full follow-up telephone interview). Women responded to a detailed questionnaire that took on average 100 minutes to complete and covered known and suspected risk factors for breast cancer, including environmental exposures, reproductive and menstrual histories, medical history, and demographic characteristics. Women were also asked about lifestyle and health behaviors across the life course, including cigarette and alcohol use [12] and recreational physical activity using a modification of the instrument developed by Bernstein et al. [15]. Data on usual dietary intake in the year prior to the baseline interview were collected using a self-completed modified Block food frequency questionnaire [16]. 

#### 2.2.2. Follow-Up Questionnaire

The full-length follow-up questionnaire, which was administered by telephone by a trained interviewer and lasted about 45 minutes, assessed treatment details for the first primary breast cancer diagnosis, factors associated with breast cancer prognosis, postdiagnosis medical history, and health behaviors. The follow-up interview included detailed questions about CAM used, specifically, before breast cancer diagnosis, after diagnosis, and during breast cancer treatment. The CAM questionnaire covered use of multivitamins, single vitamins, minerals, herbs and botanicals, other dietary supplements, mind-body practices, support groups, prayer and special diets, and visits to CAM practitioners [14].  Table 1 lists the single agents and modalities within each category.

#### 2.2.3. Medical Records

Medical records obtained at the time of baseline and follow-up were used to confirm disease characteristics and treatment course. For case women for whom complete medical records were available (*n* = 598), information abstracted from the records was in excellent agreement (*κ* = 0.92–0.96) with the women's responses to questions about treatment in the follow-up questionnaire; we therefore relied on the follow-up questionnaire data for information about cancer treatment [13].

### 2.3. Data Analysis

#### 2.3.1. Data Categorization

In this analysis, we defined self-care as use of multivitamins, single vitamins, botanicals, other dietary supplements, mind-body practices, prayer, support groups, and use of special diets. We distinguished self-care from visits to CAM practitioners, which usually entail a time commitment, an encounter, and a fee for the services of another individual. 

Within each category or type of self-care and practitioner-based CAM, we grouped the women into 4 distinct groups by timing of use in relation to their breast cancer diagnosis: “continuous users” used ≥1 modality within the category both before and after diagnosis; “starters” started using ≥1 modality from within a category after diagnosis and had never used anything from that category before diagnosis; “quitters” stopped using everything they had previously used in that category after diagnosis; and “never users” never used any modality within a category. 

#### 2.3.2. Statistical Analyses

First, univariate regression analyses were performed to identify the variables that were independently associated with use of self-care/practitioner-based CAM before and after diagnosis. Then, 2 sets of multivariable logistic regression models were developed to identify factors independently associated with (1) continuously using and (2) starting to use a category of modality after diagnosis [17]. Age, income, education, and stage at diagnosis as reported at the baseline interview, were selected as a priori confounders. All factors found to be statistically associated with always using CAM before and after diagnosis (with a *P* value of <0.05 in at least one of the categories) were included in the full models so that the odds ratios would be comparable across all models. The independent variables tested in univariate regression models that were not included in the final multivariable models (*P* > 0.05) were oral contraceptive use, hormone replacement therapy, stage at diagnosis, and hormone receptor status. The final models were adjusted for age at diagnosis, race, income, education, body mass index (BMI, defined as weight in kilograms divided by height in meters squared), health behaviors (including mammogram within 5 years prior to diagnosis, cigarette smoking, alcohol use, physical activity, and fruit and vegetable intake) as reported at baseline, continuous use of other CAM categories, and first course of breast cancer treatment type, as reported at the follow-up interview. 

## 3. Results

Among LIBCSP cases, 764 completed the full follow-up questionnaire and were included in this analysis. Differences between them and the 724 who did not have full follow-up data have been previously reported [14]. Among those included in this analysis (*n* = 764), mean age at diagnosis was 56.3 years; 94% were non-Hispanic white; 59% had an annual household income above $50,000; 61% had attended college; and 44.1% had never smoked cigarettes. At baseline, 36.8% ate at least 35 servings of fruits and vegetables per week, and 23.7% exercised at least 2.7 hours a week (Table 2).

### 3.1. Use of Self-Care and Practitioner-Based CAM before and after Diagnosis

More than 95% of our study participants used self-care after diagnosis; most did so before as well. About 75% used a multi- or single vitamin, and about 40% used prayer, botanicals, or practitioner-based CAM (data not shown).

#### 3.1.1. Continuous Users

Both before and after diagnosis, 55.5% of women used multivitamins; 57.3% used single vitamins; 35.5% used prayer, 29.2% botanicals, 21.6% special diets, and 16.6% mind-body practices. 25.4% of women used at least one practitioner-based modality both before and after diagnosis (Table 3). 

#### 3.1.2. Starters

67.2% of women started some form of self-care after diagnosis. The modalities most commonly started after diagnosis were multivitamins (20.5%), single vitamins (19.1%), support groups (17.4%), other dietary supplements (12.3%), and mind-body practices (11.6%). 14.8% of women began using a practitioner-based modality after diagnosis. The particular modalities most commonly initiated after breast cancer diagnosis were calcium (25.3%), vitamin E (22.6%), green tea (20.9%), support groups (17.4%), vitamin C (16.5%), low-fat diet (11.0%), and glucosamine (10.3%) (Table 3).

#### 3.1.3. Quitters

10.6% of women stopped using multivitamins after diagnosis. The most common modalities stopped after diagnosis were iron (11.9%) and vitamin C (9.3%). A small proportion of women (7.2%) stopped using all practitioner-based modalities. Chiropractic, the most common ever-used practitioner-based modality (210 users or 27.5%), was the modality most commonly discontinued after diagnosis (14.5%) (Table 3). 

### 3.2. CAM Use and Self-Care during Adjuvant Treatment 

#### 3.2.1. Use during Treatment

As previously reported [14], all 764 respondents who participated in the complete follow-up study reported undergoing surgery for their breast cancer; 310 (40.6%) also received chemotherapy, 464 (60.7%) radiation therapy, and 462 (60.5%) tamoxifen treatment. The forms of self-care most frequently used during chemotherapy were prayer (48.7%), multivitamins (37.42%), single vitamins (33.2%), mind-body (28.4%), and support groups (23.9%); 21.6% of women used practitioner-based modalities. The forms of self-care most commonly used during radiation therapy were multivitamins (55.2%), single vitamins (48.7%), prayer (39.9%), mind-body (31.0%), and special diets (29.0%); 17.4% of women used practitioner-based modalities. The self-care modalities most commonly used during tamoxifen treatment were multivitamins (80.7%), prayer (38.1%), single vitamins (23.6%), mind-body (22.9%), and special diets (26.0%); 26.0% of women used practitioner-based modalities (Table 4).

### 3.3. Factors Associated with Self-Care and Practitioner-Based CAM before and after Diagnosis

#### 3.3.1. Demographics

Higher education was a strong predictor of both starting mind-body practices after diagnosis and using such practices before and after diagnosis (continuous use); education was also associated with continuous use of botanicals and dietary supplements. Income was associated directly with continuous single vitamin use and inversely with continuous use of other dietary supplements (Table 5); income was inversely associated with beginning practitioner-based CAM after diagnosis (Table 6). Race was the demographic variable most strongly associated with starting self-care after diagnosis; women of minority race/ethnicity were about 3.5 times as likely to start using botanicals or special diets as non-Hispanic whites (Table 6). Younger women and women with more education were more likely than older and less educated women to start using noningestible forms of self-care or practitioner-based CAM after diagnosis (Table 6).

#### 3.3.2. Health Behaviors

Healthy behaviors reported at the baseline interview were more strongly associated with continuously using self-care than with starting self-care after a diagnosis with first primary breast cancer. Those with the highest level of physical activity (>2.7 hr/week) were 2.4 times as likely to use mind-body modalities before and after diagnosis as those who reported no physical activity. Women who had a mammogram within 5 years before diagnosis were 2 times as likely to be continuous users of single vitamins as women who had not had a recent mammogram. Women whose intake of fruits and vegetables was high were 50% more likely to use multivitamins and/or botanicals continuously than those whose intake was low (Table 5).

Current smokers had 60% lower odds of following a special diet before and after diagnosis than never smokers (Table 5). Women with a body mass index (BMI) ≥30 had 60% lower odds of always using other dietary supplements and women with a BMI between 25–29 had 50% lower odds of continuously using mind-body practices than women with a BMI <25. Women with BMI ≥30 were twice as likely to start mind-body practices as women with a BMI <25 but nearly 80% less likely to start a special diet (Table 6). 

High BMI was also associated with continuous use of practitioner-based modalities (OR = 2.5); high fruit and vegetable intake was associated with starting practitioner-based CAM after diagnosis (OR = 1.8). 

#### 3.3.3. Continuous Use of Other Self-Care and Practitioner-Based CAM

In our multivariable logistic regression models, among women with a diagnosis of first primary breast cancer, the strongest and most common predictors of continuous use of one type of self-care were continuous use of other types of self-care. In particular, users of one type of ingestible self-care were more likely to use other ingestible agents. Women who continuously used single vitamins were nearly 4 times as likely to use multivitamins and/or use other dietary supplements and 1.6 times as likely to use botanicals before and after diagnosis as other women. Similarly, women who used botanicals before and after diagnosis were nearly twice as likely to use multivitamins and single vitamins before and after diagnosis as never users of botanicals (Table 5).

Women who used ingestible forms of self-care were also more likely to begin using new categories of ingestible self-care after diagnosis with first primary breast cancer. Women who used single vitamins before and after diagnosis were 10 times as likely to start use of botanicals after diagnosis as never users; women who used other dietary supplements before and after diagnosis were 3 times more likely to start using botanicals after diagnosis than never users (Table 6).

Continuous users of mind-body modalities were more than 7 times as likely to use prayer, 5 times as likely to use support groups, and more than twice as likely to use practitioner-based modalities continuously as non-users. 

#### 3.3.4. Disease Characteristics

Neither stage of disease nor hormone receptor status was associated with continuous or new use of self-care categories. The only clinical variable associated with self-care was treatment type: women who received tamoxifen treatment had 1.7 times greater odds of starting use of single vitamins after diagnosis than women who were not treated with tamoxifen (Table 6). 

## 4. Discussion

We present a novel method to describe changes in CAM use before and after cancer diagnosis by comparing those who started using, stopped using, continued using, or never used a category of CAM following diagnosis. Cancer survivors access multiple forms of CAM and we specifically examined differences in patterns of CAM that involved “self-care” as compared to those that were based on “practitioner-care.” Among women diagnosed with a first primary breast cancer who self-reported their use of complementary and alternative medicine modalities in the follow-up components of the Long Island Breast Cancer Study Project, the factors most commonly and strongly associated with using self-care and CAM modalities were use of other categories of self-care. In particular, women who used one form of ingestible self-care (multi- and single vitamins, botanicals, other dietary supplements, or special diets) were more likely than other women either to continue or to start using other ingestible self-care. Healthy behaviors were associated with self-care practices that were used before diagnosis and continued afterwards. Behavioral factors were more predictive of self-care use after breast cancer diagnosis than were indicators of disease prognosis.

In a nationally representative survey conducted in 1997, which is close to the period when the LIBCSP cases were diagnosed with breast cancer, 42% of adults reported using at least 1 of 16 CAM modalities (these data did not include daily vitamins) [2]. Of those who used CAM, 46% reported visiting a CAM practitioner within the past year [2]. The prevalence of CAM practitioner visits among the 1997 sample is comparable to that among the LIBCSP cases. Among the adults surveyed in the 2002 National Health Interview Survey, 62% of adults reported using CAM, although this dropped to 36% when health-related prayer was excluded; natural products and breathing/meditation were the most commonly used forms of CAM [3]. 95% of LIBCSP case women included in this analysis used self-care after breast cancer diagnosis, and 90% used self-care after diagnosis if multivitamins are excluded, which is much higher than in national samples of adults.

A recent cross-sectional study reported that 75% of adult cancer patients used CAM and of those, close to 60% began using CAM after their cancer diagnosis [18]. It has been well documented that women with breast cancer who use CAM are younger, better educated, and more affluent than women with breast cancer who do not use CAM [2, 19–23]; our results are consistent with these findings. In the Women's Healthy Eating and Living Study, 81% of breast cancer survivors reported using dietary supplements [24]. In the Pathways Study, 29% of breast cancer patients diagnosed in 2008-2009 in Northern California used special diets after diagnosis, as compared with 33% of LIBCSP women. However, more of the participants of the Pathways Study than of the LIBCSP used botanicals (48% versus 40%), mind-body healing (including support groups) (64% versus 49%), and other dietary supplements (47% versus 25%) after diagnosis [9]. Differences in location and period of recruitment may account for the differences in CAM use in the two samples. 

It is not surprising that the strongest predictor overall of using a self-care category before and after diagnosis with a first primary breast cancer, and of starting a self-care category after diagnosis, was using other forms of self-care. Women who already use some form of CAM or a self-care practice are likely to be aware of other CAM modalities. It is also not surprising that ingestible forms of CAM were commonly used together. Women who already take one type of supplement do not need to make much additional effort or to undergo a significant change in behavior to try new supplements. Continuous use of botanicals was the factor most commonly associated with starting new forms of self-care, including single vitamins, mind-body, other dietary supplements, and special diets. However, starting practitioner-based CAM was not associated with prior use of self-care modalities. 

High intake of fruits and vegetables at the baseline interview was associated with the use of multivitamins before and after diagnosis. High recreational physical activity level reported at the baseline interview was positively associated with continuous use of mind-body practices, and high BMI at baseline was negatively associated with continuous use of botanicals, other dietary supplements, and mind-body modalities. However, healthy behaviors were not strongly associated with starting self-care practices after diagnosis; most women who engaged in healthy behaviors were already using self-care. 

Neither stage of disease nor hormone receptor status was associated with starting either self-care or practitioner-based modalities. Most self-care and practitioner-based CAM use occurred during tamoxifen treatment, perhaps because tamoxifen is an oral agent that patients use for 5 years, whereas chemotherapy and radiation treatments have a usual duration of several months or weeks. In addition, women who used tamoxifen may have been more willing to use other oral or ingestible agents than women who did not use tamoxifen. Mind-body practices were also common during adjuvant treatment, perhaps because women who were told not to take dietary supplements during those treatments used noningestible forms of self-care instead. 

The modalities that were most commonly initiated after diagnosis with breast cancer among women in our study were also the modalities most frequently used during adjuvant treatment. Although our study participants were not asked why they used the modalities they chose, data from other studies suggest that patients initiate self-care after breast cancer diagnosis due to fear or experience of treatment-related side effects [25–27]. A recent study that examined CAM use among breast cancer patients after adjuvant therapy similarly compared self-care (or “self-directed”) CAM with “provider-directed” CAM [28]. Of those women who used self-directed CAM, over half reported using it to “influence the course of cancer after adjuvant therapy,” while 95% of women who used self-directed CAM and all women who used provider-directed CAM used it to improve wellbeing [28]. This study highlights the importance of discussing CAM use with breast cancer patients, particularly in the context of adjuvant therapy. 

Among the limitations of our study, the follow-up data were collected nearly a decade ago; CAM and self-care practices may be different now. And because the data were collected more than 5 years after diagnosis and treatment, patients may not have accurately recalled their prior CAM use. Although minority race/ethnicity was associated with beginning practitioner-based CAM, only 6% of the sample were non-White, and thus the associations observed are unstable and must be interpreted with caution. In addition, although two-thirds of the case women who participated in the original LIBCSP case-control study provided some follow-up data, only 55.4% personally completed the full follow-up questionnaire, including the instrument developed by our team to assess use of CAM. We previously reported that nonrespondents to the full follow-up were of lower socioeconomic status than respondents (response bias) [14]. The well-established association of higher income with CAM use may partially explain the high prevalence of CAM use in our study population. 

Growing evidence suggests that CAM and other self-care modalities may be effective in reducing treatment-related side effects, including nausea and vomiting, as well as in reducing stress and easing pain [26, 29]. Further, engagement in self-care practices helps individuals with cancer to maintain some sense of control over their personal wellbeing. Self-efficacy and feelings of control are predictive of improved coping, emotional wellbeing, physical health, immune function, and quality of life among cancer patients [30–33]. Henderson and Donatelle found that higher perceptions of control predicted CAM use among breast cancer patients [11]. The connection between self-care during cancer and a variety of physical and psychological outcomes should be further explored.

## 5. Conclusions

We found that a diagnosis of breast cancer was associated with subsequent behavioral changes and that more patients undertook new forms of self-care than abandoned the forms they had been using. The most popular modalities were those that are ingestible and readily available without a gatekeeper, and they were commonly used in combination. These findings are important because they identify patterns of CAM use in a well-characterized population of cancer patients, including how CAM use changed after diagnosis and during treatment. Based on research conducted to date, evidence-based guidelines are available for providers and patients about the safety and effects of various CAM modalities in the oncology setting [34–36] and can inform how and when clinicians counsel their patients on CAM use. However, more research is needed to understand the specific effects of many CAM therapies on cancer outcomes and quality of life, and further research is needed to understand the motivations for and patterns of CAM use so that a broader spectrum of patient needs can be met.

## Figures and Tables

**Table 1:** List of individual modalities within each category of self-care and practitioner-based CAM, reported in the LIBCSP follow-up interview (2002–2004).

Category	Individual modalities
Multivitamins	Multivitamin with minerals, Multivitamin without minerals, antioxidant combination type (A, C, E), stress-tabs, women's formula (MV), multivitamin with Herbs, other multi-vitamin

Single vitamins	Vitamin A with beta carotene, vitamin A without beta carotene, beta carotene (alone), vitamin B1/thiamin, vitamin B3/niacin, vitamin B6, vitamin B12, B complex vitamins, vitamin C, vitamin D and calcium, vitamin D, vitamin E (alpha-tocopherol), magnesium, calcium, rolaids, tums, dolomite, folic acid/folate, selenium, iron, zinc, lutein, chromium

Botanicals	*Aloe vera*, *Ashwagandha*, *Atractylodes*, *Astragalus* root, Bee pollen, *Bilberry*, Black cohosh, clue cohosh, blue-green algae, borage seed oil, bromelain, *Burdock*, *Calendula*, Cascara sagrada, cat's claw, chamomile, chaste tree, Vitex, or chaste berry, cranberry, *Dandelion*, dong quai, *Echinacea*, elder, ephedra, essiac or florEssence, evening primrose oil, *Fennel*, feverfew, garlic, ginger, *Ginkgo biloba*, ginseng, goldenseal, grape seed oil, green tea, hawthorne, horse chestnut, kava kava, kelp, lavender, maitake mushroom, milk thistle, *Nettle*, pau d'arco, proanthocyanidin, pycnogenol, red clover, reishi mushroom, shiitake mushroom, slippery elm, soy supplements or isoflavones, St. John's Wort, valerian, wild yam, willow bark

Other dietary supplements Products	Fiber supplement, coenzyme Q10 (CoQ10), shark cartilage, melatonin, flax seed oil, fish oil/EPA/Omega-3/cod liver oil, glucosamine, chondroitin, DHEA, acidophilus, arginine, leucine

Mind-body	Meditation, visualization/imagery, tai chi, qi gong, yoga, dance therapy, art therapy, music therapy, poetry therapy or journaling, biofeedback

Special diets	Vegan diet, vegetarian diet, no red meat, organic fruits and vegetables, macrobiotic diet, low-fat diet, high-fiber diet, changed your consumption of soy products, diet or program designed to lose weight

Practitioner-based	Massage, water treatment or hydrotherapy, reiki, healing touch, or other energy therapy, bioeletromagnetic therapy, acupuncture, ayurvedic medicine, Chinese medicine, chiropractic therapy, herbalist, homeopathy, native american Medicine, naturopathic physician, nutritionist or dietician, tibetan, other CAM practitioner, hypnosis, psychotherapy

**Table 2:** Demographics, health behaviors, and disease characteristics, by category of self-care and practitioner-based CAM used after diagnosis among women in the LIBCSP.

	TOTAL (*n* = 764)	Multivitamins^b^ (*n* = 581, 76%)	Single vitamins/minerals^b^ (*n* = 584, 76.4%)	Botanicals^b^ (*n* = 303, 39.7%)	Other dietary supplements^b^ (*n* = 188, 24.6%)	Mind-body^b^ (*n* = 216, 28.3%)	Special diets^b ^(*n* = 248, 32.5%)	Prayer^b^ (*n* = 302, 39.5%)	Support groups^b^ (*n* = 161, 21.1%)	CAM practitioners^b ^ (*n* = 307, 40.2%)
Demographics										
Age at diagnosis^a^										
Continuous (Mean ± SD)	56.3 ± 11.4	55.8 ± 11.2	56.1 ± 10.9	54.4 ± 10.6	55.1 ± 9.9	52.2 ± 10.1	55.9 ± 10.6	53.3 ± 10.2	51.7 ± 9.6	53.3 ± 10.7
<45	17.8%	18.6%	17.3%	21.5%	12.8%	25.9%	17.7%	21.9%	29.8%	22.8%
45–54	30.2%	31.2%	32.0%	32.3%	38.3%	35.6%	30.6%	36.1%	34.2%	37.5%
55–64	26.7%	26.9%	27.2%	29.0%	29.3%	26.9%	27.4%	27.5%	27.3%	24.4%
65+	25.3%	23.4%	23.5%	17.2%	19.7%	11.6%	24.2%	14.6%	8.7%	15.3%
Race^a^										
Non-Hispanic white	94.0%	93.5%	93.8%	93.1%	96.3%	92.6%	89.5%	92.1%	93.8%	93.8%
All other	6.0%	6.5%	6.2%	6.9%	3.7%	7.4%	10.5%	7.9%	6.2%	6.2%
Religion^a,c^										
Protestant	22.6%	23.1%	21.1%	20.5%	25.0%	24.1%	21.4%	22.2%	24.2%	20.5%
Catholic	55.5%	55.1%	55.5%	54.5%	48.4%	55.6%	50.8%	64.2%	53.4%	54.1%
Jewish	20.0%	20.5%	21.7%	23.8%	24.5%	19.0%	25.4%	12.3%	21.7%	23.1%
Annual household income^a^										
<$25k	10.3%	9.3%	7.5%	6.9%	8.0%	7.9%	10.5%	8.6%	3.7%	9.1%
$25k–$49,999	30.6%	30.6%	30.0%	25.7%	24.5%	25.0%	26.2%	30.1%	24.8%	21.5%
$50k–$89,999	34.8%	35.6%	36.0%	38.0%	38.3%	40.7%	34.7%	38.7%	37.9%	37.8%
≥$90k	24.2%	24.4%	26.5%	29.4%	29.3%	26.4%	28.6%	22.5%	33.5%	31.6%
Education^a^										
≤High school grad	39.4%	36.3%	35.8%	26.1%	27.1%	20.8%	32.3%	32.8%	23.0%	28.3%
Some college	25.5%	26.7%	26.0%	27.7%	26.1%	30.6%	27.4%	29.8%	24.8%	25.4%
College graduate	14.5%	15.0%	15.2%	18.5%	15.4%	15.7%	16.5%	13.9%	23.0%	18.9%
Postgraduate	20.5%	22.0%	22.9%	27.7%	31.4%	32.9%	23.8%	23.5%	29.2%	27.4%
BMI at diagnosis^a^										
<25	49.1%	51.3%	51.0%	53.8%	53.7%	56.0%	52.0%	52.6%	50.9%	48.2%
25–29	31.5%	30.8%	31.8%	30.7%	30.3%	25.0%	32.3%	27.2%	32.3%	30.6%
≥30	19.4%	17.9%	17.1%	15.5%	16.0%	19.0%	15.7%	20.2%	16.8%	21.2%
Menopausal status^a^										
Premenopausal	36.5%	37.7%	37.8%	43.2%	34.6%	52.3%	38.7%	45.4%	50.9%	46.6%
Postmenopausal	60.9%	59.7%	59.4%	53.8%	60.6%	44.4%	57.7%	51.3%	47.8%	49.8%
Health behaviors										
Oral contraceptives^a^										
Never	49.2%	47.3%	47.6%	46.5%	44.1%	38.9%	47.6%	45.4%	36.6%	43.0%
Ever	50.5%	52.5%	52.1%	53.5%	55.3%	60.6%	52.0%	54.6%	63.4%	57.0%
Hormone replacement therapy^a^										
Never	66.9%	67.1%	65.1%	64.0%	52.7%	67.6%	64.1%	68.2%	64.6%	65.5%
Ever	33.1%	32.9%	34.9%	36.0%	47.3%	32.4%	35.9%	31.8%	35.4%	34.5%
Mammogram^a^										
Never or >5 yrs ago	4.2%	4.1%	3.3%	3.0%	^ d^	3.7%	3.2%	4.3%	5.0%	4.2%
<5 yrs ago	94.4%	94.3%	95.4%	96.0%	96.8%	96.3%	96.8%	95.4%	94.4%	94.8%
Cigarette smoking status^a^										
Never	44.1%	44.9%	45.9%	48.2%	45.7%	47.7%	48.8%	49.0%	49.1%	45.0%
Current	18.6%	17.2%	17.0%	18.2%	14.4%	15.7%	11.3%	17.5%	13.7%	17.6%
Former	37.3%	37.9%	37.2%	33.7%	39.9%	36.6%	39.9%	33.4%	37.3%	37.5%
Alcohol use^a^										
Never	33.8%	33.7%	31.8%	31.0%	30.9%	33.3%	33.9%	34.1%	32.3%	33.9%
Ever	66.2%	66.3%	68.2%	69.0%	69.1%	66.7%	66.1%	65.9%	67.7%	66.1%
Average lifetime alcohol intake (g/day)^a^										
0	34.2%	34.1%	32.4%	31.4%	31.4%	34.3%	34.3%	34.8%	32.9%	34.5%
<15	49.3%	50.8%	51.2%	52.5%	47.3%	47.7%	48.0%	50.0%	46.0%	51.5%
15–30	11.6%	11.0%	11.8%	11.6%	12.8%	11.6%	12.1%	10.3%	11.8%	9.4%
≥30	4.8%	4.1%	4.6%	4.6%	8.5%	6.5%	5.6%	5.0%	9.3%	4.6%
Physical activity (h/wk)^a^										
0	24.2%	22.9%	22.3%	17.5%	18.6%	13.4%	16.1%	19.2%	17.4%	19.2%
0–0.69	22.5%	21.3%	22.1%	22.4%	23.9%	23.1%	23.8%	22.5%	18.6%	20.8%
0.70–2.6	24.1%	26.2%	25.2%	24.4%	22.9%	26.9%	24.6%	24.8%	29.2%	24.1%
≥2.7	23.7%	24.3%	24.8%	29.7%	27.7%	31.5%	29.8%	27.2%	26.7%	29.3%
Fruit and vegetable intake (servings/wk)^a^										
0–34	62.2%	60.1%	61.0%	54.5%	57.4%	55.6%	57.3%	58.3%	55.3%	57.7%
≥35	36.8%	38.7%	37.8%	44.6%	41.5%	44.0%	42.7%	40.7%	42.9%	41.4%
CAM Use before diagnosis^b^										
Multivitamins	55.5%	73.0%	62.8%	70.3%	66.5%	64.8%	60.1%	62.9%	64.0%	61.2%
Single vitamins	57.3%	64.2%	75.0%	75.6%	74.5%	68.1%	66.5%	63.6%	67.7%	67.8%
Botanicals	29.2%	33.7%	34.8%	73.6%	40.4%	42.1%	40.7%	39.7%	38.5%	40.4%
Other diet. supp.	12.3%	14.1%	14.6%	19.1%	50.0%	24.1%	16.1%	16.9%	23.0%	20.8%
Mind-body	16.6%	18.8%	18.2%	24.4%	30.9%	58.8%	24.2%	33.4%	35.4%	26.1%
Special diets	21.6%	21.9%	23.5%	29.0%	30.9%	33.8%	66.5%	27.5%	28.6%	27.4%
CAM practitioner	25.4%	27.7%	28.8%	38.3%	40.4%	45.4%	33.1%	36.4%	43.5%	63.2%
Disease characteristics										
Stage at diagnosis^a^										
In situ	17.9%	18.8%	18.7%	20.1%	23.4%	19.4%	20.6%	18.5%	14.3%	21.2%
Invasive	82.1%	81.2%	81.3%	79.9%	76.6%	80.6%	79.4%	81.5%	85.7%	78.8%
Hormone receptor status^a^										
ER−/PR−	13.0%	13.3%	12.3%	13.5%	11.2%	12.0%	12.5%	14.6%	12.4%	10.1%
ER+ or PR+	49.2%	48.2%	46.1%	45.9%	43.1%	50.5%	47.2%	47.7%	52.2%	50.2%
missing	37.8%	38.6%	41.6%	40.6%	45.7%	37.5%	40.3%	37.7%	35.4%	39.7%
Treatment received^b^										
Chemotherapy	40.6%	40.6%	39.7%	45.9%	38.3%	51.4%	43.1%	48.3%	57.1%	44.0%
Radiation	60.7%	61.4%	61.8%	60.4%	59.6%	61.1%	57.3%	59.9%	55.9%	58.0%
Tamoxifen	60.5%	60.6%	59.6%	55.8%	57.4%	58.8%	60.1%	60.9%	60.2%	55.4%

^a^Data collected at baseline, 1996-1997.

^b^Data collected at follow-up, 2002–2004.

^c^Data for no religion/other religion are not displayed because cell size <5.

^d^Data are not displayed because cell size <5.

**Table 3:** Timing of the top 6 self-care and practitioner based modalities per category among women in the LIBCSP follow-up interview (2002–2004).

	Starters	Continuers	Quitters	Never users
Multivitamins (any)	20.5%	55.5%	10.6%	13.4%
Single Vitamins	19.1%	57.3%	5.0%	18.6%
Calcium	25.3%	20.9%	4.1%	49.7%
Vitamin E	22.6%	24.7%	4.2%	48.4%
Vitamin C	16.5%	32.7%	9.3%	41.5%
B12	8.9%	3.5%	5.2%	82.3%
B Complex vitamins	7.9%	7.1%	4.5%	80.6%
Iron	3.7%	2.1%	11.9%	82.3%
Botanicals	10.5%	29.2%	1.6%	58.8%
Green tea	20.9%	2.4%	^ a^	76.3%
*Echinacea *	9.8%	4.7%	1.2%	84.3%
*Ginkgo biloba *	4.6%	1.6%	1.3%	92.5%
Black Cohosh	2.1%	^ a^	^ a^	97.5%
St. John's Wort	1.3%	^ a^	^ a^	98.2%
Chamomile	0.7%	1.6%	0.0%	97.8%
Other OTC products	12.3%	12.3%	3.0%	72.4%
Glucosamine	10.3%	^ a^	0.7%	88.5%
Chondroitin	8.8%	^ a^	0.9%	89.8%
Co-Q-10	8.1%	2.0%	^ a^	89.4%
Fish oil	5.5%	2.2%	3.3%	89.0%
Fiber supplements	4.7%	5.0%	3.0%	87.3%
Mind-body	11.6%	16.6%	1.4%	70.3%
Visualization	7.7%	4.5%	0.7%	83.2%
Meditation	6.9%	8.5%	1.2%	83.2%
Yoga	6.8%	3.3%	1.7%	83.2%
Tai chi	3.3%	0.7%	1.4%	83.2%
Poetry	2.6%	3.1%	^ a^	83.2%
Music therapy	1.8%	5.2%	0.0%	83.2%
Prayer	4.80%	35.5%	0.0%	59.7%
Support groups	17.40%	3.70%	0.8%	78.1%
Special diets	10.9%	21.6%	1.0%	66.5%
Low-fat diet	11.0%	14.9%	1.0%	72.1%
Weight loss	8.8%	4.1%	3.5%	83.1%
Change consumption of soy	6.9%	1.2%	^ a^	91.6%
No red meat	6.4%	3.8%	^ a^	89.3%
Organic	5.9%	1.4%	^ a^	92.5%
High fiber	5.1%	3.0%	^ a^	91.6%
Practitioner-based	14.8%	25.4%	7.2%	52.6%
Massage	9.4%	7.6%	0.7%	81.9%
Chiropractor	6.2%	12.0%	8.9%	72.5%
Acupuncture	6.0%	1.4%	2.8%	89.7%
Reiki, healing touch, other energy	5.5%	0.9%	^ a^	92.9%
psychotherapy	5.5%	8.1%	2.1%	84.3%
Nutritionist or dietician	3.5%	^ a^	0.7%	95.7%

^a^Data are not displayed due to cell sizes <5.

**Table 4:** Proportion of CAM use during treatment, by treatment type, among women in the LIBCSP follow-up interview (2002–2004).

	Chemotherapy (*n* = 310)	Radiation (*n* = 464)	Tamoxifen (*n* = 462)
Multivitamins (any)	37.4%	55.2%	80.7%
Single vitamins	33.2%	48.7%	54.1%
Vitamin E	18.1%	18.8%	34.6%
Vitamin C	18.1%	19.0%	32.3%
Calcium	12.3%	12.3%	28.8%
Selenium	6.5%	6.3%	8.4%
B-complex	4.8%	4.1%	8.2%
Herbs and botanicals	13.2%	12.6%	23.6%
Green tea	8.1%	4.3%	13.6%
*Echinacea *	3.5%	1.1%	7.6%
*Ginkgo biloba *	^ a^	^ a^	3.7%
Chamomile	^ a^	^ a^	1.5%
Garlic	^ a^	^ a^	1.1%
Black cohosh	0.0%	0.0%	1.1%
*Astragalus* root	^ a^	^ a^	^ a^
Other OTC Products	8.1%	10.0%	16.2%
Glucosamine	0.0%	^ a^	6.3%
Co-Q-10	2.3%	2.4%	5.6%
Chondroitin	0.0%	^ a^	5.4%
Laxatives	3.2%	1.9%	5.4%
Flax seed oil	^ a^	^ a^	3.7%
Fish oil/EPA/Omega-3	^ a^	1.5%	3.5%
Melatonin	^ a^	^ a^	2.2%
Mind-body	28.4%	31.0%	22.9%
Meditation	16.1%	12.3%	13.0%
Visualization	14.5%	9.9%	8.0%
Yoga	^ a^	1.5%	6.9%
Music therapy	8.7%	5.8%	6.3%
Poetry therapy or journaling	7.1%	3.9%	3.7%
Tai chi	1.6%	^ a^	2.6%
Support groups	23.9%	11.2%	12.8%
Prayer	48.7%	39.9%	38.1%
Practitioner based	21.6%	17.4%	26.0%
Chiropractor	4.5%	2.8%	11.3%
Psychotherapy	11.3%	5.8%	9.1%
Massage	5.5%	1.9%	8.7%
Reiki	4.2%	1.7%	3.7%
Acupuncture	^ a^	^ a^	2.6%
Nutritionist	2.6%	1.3%	1.5%
Special diets	22.9%	29.0%	26.0%
Low-fat	17.4%	15.9%	22.1%
No red meat	7.4%	6.5%	8.0%
Weight loss	1.6%	2.6%	8.0%
High fiber diet	5.2%	4.5%	5.8%
Organic fruits and vegetables	6.8%	4.7%	4.3%
Changed consumption of soy	4.2%	3.0%	3.7%

^a^Data are not displayed due to cell sizes <5.

**Table 5:** Factors associated with *continuing* (vs. starting, stopping, or never using) self-care and practitioner-based CAM among women in the LIBCSP, multivariable analyses, OR (95% Confidence Interval).

	Multivitamins^b^	Single vitamins/minerals^b^	Botanicals^b^	Other dietary supplements^b^	Mind-body^b^	Special diets^b^	Prayer^b^	Support groups^b^	CAM practitioners^b^
Age^a^									
Continuous	0.98 (0.95–1.00)	1.01 (0.98–1.04)	1.00 (0.98–1.03)	**0.98 (0.94–1.02**)	1.00 (0.97–1.04)	1.00 (0.97–1.03)	**0.97 (0.94–1.00)**	1.01 (0.93–1.08)	0.99 (0.96–1.02)
Race^a^									
Non-Hispanic white	1 (Ref)	1 (Ref)	1 (Ref)	1 (Ref)	1 (Ref)	1 (Ref)	1 (Ref)	1 (Ref)	1 (Ref)
All Other	0.78 (0.39–1.60)	1.09 (0.50–2.35)	0.60 (0.26–1.36)	0.40 (0.10–1.62)	1.89 (0.80–4.49)	**2.18 (1.05–4.53)**	1.10 (0.52–2.32)	1.74 (0.30–10.11)	0.95 (0.40–2.22)
Annual household income^a^									
<$25k	1 (Ref)	1 (Ref)	1 (Ref)	1 (Ref)	1 (Ref)	1 (Ref)	1 (Ref)	1 (Ref)	1 (Ref)
$25k–$49,999	1.14 (0.60–2.14)	**2.37 (1.21–4.64)**	1.38 (0.64–2.98)	0.38 (0.13–1.10)	0.78 (0.33–1.83)	0.56 (0.26–1.19)	1.79 (0.86–3.72)	—	0.76 (0.34–1.72)
$50k–$89,999	0.97 (0.50–1.91)	**2.61 (1.28–5.30)**	1.12 (0.50–2.50)	0.40 (0.13–1.19)	0.50 (0.20–1.25)	0.73 (0.33–1.59)	1.38 (0.64–2.97)	—	0.79 (0.34–1.82)
≥$90k	0.87 (0.42–1.82)	2.09 (0.96–4.55)	1.11 (0.47–2.60)	0.31 (0.10–1.01)	0.45 (0.16–1.21)	0.96 (0.42–2.22)	1.00 (0.43–2.31)	—	0.82 (0.33–2.02)
Education^a^									
≤High school grad	1 (Ref)	1 (Ref)	1 (Ref)	1 (Ref)	1 (Ref)	1 (Ref)	1 (Ref)	1 (Ref)	1 (Ref)
Some college	1.25 (0.80–1.96)	1.00 (0.64–1.58)	**1.82 (1.12–2.95)**	0.86 (0.38–1.99)	**2.38 (1.29–4.39)**	1.26 (0.74–2.15)	1.02 (0.64–1.64)	0.91 (0.24–3.40)	1.26 (0.73–2.17)
College graduate	1.17 (0.66–2.08)	1.00 (0.55–1.83)	**2.72 (1.49–4.95)**	2.17 (0.88–5.39)	1.36 (0.60–3.08)	1.25 (0.65–2.42)	0.76 (0.41–1.41)	1.31 (0.29–5.98)	1.83 (0.96–3.51)
Postgraduate	0.85 (0.50–1.45)	1.12 (0.64–1.96)	**2.25 (1.28–3.95)**	2.60 (1.13–5.97)	**2.44 (1.20–4.99)**	1.07 (0.58–1.98)	0.80 (0.45–1.42)	0.73 (0.17–3.14)	1.54 (0.83–2.84)
BMI at diagnosis^a^									
<25	1 (Ref)	1 (Ref)	1 (Ref)	1 (Ref)	1 (Ref)	1 (Ref)	1 (Ref)	1 (Ref)	1 (Ref)
25–29	0.79 (0.53–1.17)	1.10 (0.73–1.66)	1.00 (0.66–1.52)	0.74 (0.39–1.40)	**0.53 (0.30–0.91)**	1.21 (0.77–1.90)	0.96 (0.63–1.47)	0.74 (0.21–2.63)	1.58 (0.99–2.53)
≥30	0.78 (0.48–1.25)	0.76 (0.46–1.26)	0.58 (0.34–1.01)	**0.40 (0.17–0.98)**	0.76 (0.40–1.46)	1.11 (0.63–1.97)	1.27 (0.76–2.10)	1.90 (0.58–6.21)	**2.52 (1.44–4.43)**
Menopausal (meno.) status^a^									
Premeno.	1 (Ref)	1 (Ref)	1 (Ref)	1 (Ref)	1 (Ref)	1 (Ref)	1 (Ref)	1 (Ref)	1 (Ref)
Postmeno.	1.34 (0.80–2.24)	0.86 (0.50–1.47)	0.94 (0.55–1.61)	2.81 (1.27–6.24)	0.94 (0.48–1.84)	0.98 (0.54–1.78)	1.03 (0.60–1.77)	0.70 (0.18–2.74)	0.66 (0.37–1.18)
Mammogram^a^									
Never/>5 yrs ago	1 (Ref)	1 (Ref)	1 (Ref)	1 (Ref)	1 (Ref)	1 (Ref)	1 (Ref)	1 (Ref)	1 (Ref)
<5 yrs ago	0.85 (0.52–1.37)	**1.95 (1.13–3.38)**	1.10 (0.64–1.91)	2.26 (0.64–7.92)	1.43 (0.61–3.34)	2.01 (0.76–5.32)	1.09 (0.65–1.82)	0.85 (0.24–3.04)	0.70 (0.41–1.19)
Physical activity (h/wk)^a^									
0	1 (Ref)	1 (Ref)	1 (Ref)	1 (Ref)	1 (Ref)	1 (Ref)	1 (Ref)	1 (Ref)	1 (Ref)
0–0.69	1.41 (0.87–2.30)	0.68 (0.41–1.12)	1.51 (0.87–2.61)	1.30 (0.55–3.05)	1.80 (0.86–3.79)	1.64 (0.90–2.97)	1.09 (0.64–1.86)	0.66 (0.14–3.04)	1.25 (0.67–2.33)
0.70–2.6	1.50 (0.93–2.43)	1.00 (0.60–1.64)	1.17 (0.68–2.01)	1.26 (0.56–2.84)	1.70 (0.82–3.50)	0.95 (0.52–1.73)	1.12 (0.67–1.89)	1.49 (0.39–5.73)	1.28 (0.71–2.32)
≥2.7	1.31 (0.80–2.14)	1.13 (0.68–1.90)	1.46 (0.85–2.50)	0.97 (0.42–2.22)	**2.44 (1.20–4.97)**	1.38 (0.76–2.48)	0.98 (0.57–1.68)	0.67 (0.14–3.13)	1.54 (0.85–2.80)
Cigarette smoking status^a^									
Never	1 (Ref)	1 (Ref)	1 (Ref)	1 (Ref)	1 (Ref)	1 (Ref)	1 (Ref)	1 (Ref)	1 (Ref)
Current	0.94 (0.58–1.52)	0.79 (0.48–1.31)	1.17 (0.70–1.96)	1.03 (0.46–2.32)	0.83 (0.41–1.70)	**0.44 (0.23–0.87)**	0.75 (0.45–1.27)	0.85 (0.16–4.63)	1.00 (0.56–1.79)
Former	1.25 (0.85–1.84)	0.82 (0.55–1.22)	0.77 (0.51–1.17)	0.66 (0.35–1.23)	1.24 (0.75–2.06)	1.10 (0.71–1.69)	0.78 (0.51–1.18)	2.16 (0.77–6.06)	1.23 (0.78–1.94)
Alcohol use^a^									
Never	1 (Ref)	1 (Ref)	1 (Ref)	1 (Ref)	1 (Ref)	1 (Ref)	1 (Ref)	1 (Ref)	1 (Ref)
Ever	1.01 (0.69–1.47)	**1.51 (1.03–2.22)**	0.79 (0.53–1.18)	1.05 (0.56–1.99)	0.97 (0.57–1.62)	1.01 (0.65–1.56)	0.91 (0.61–1.35)	0.55 (0.20–1.54)	1.43 (0.91–2.26)
Fruit and vegetable intake (servings/ wk)^a^									
0–34	1 (Ref)	1 (Ref)	1 (Ref)	1 (Ref)	1 (Ref)	1 (Ref)	1 (Ref)	1 (Ref)	1 (Ref)
≥35	**1.51 (1.06–2.17)**	1.18 (0.81–1.71)	**1.46 (1.00–2.12)**	0.87 (0.49–1.55)	1.44 (0.91–2.30)	1.23 (0.82–1.84)	1.24 (0.85–1.81)	0.69 (0.25–1.94)	0.82 (0.54–1.25)
Stage at diagnosis^a^									
In situ	(1 Ref)	(1 Ref)	(1 Ref)	(1 Ref)	(1 Ref)	(1 Ref)	(1 Ref)	(1 Ref)	(1 Ref)
Invasive	0.80 (0.48–1.34)	1.47 (0.86–2.53)	0.66 (0.38–1.13)	0.88 (0.39–1.98)	0.99 (0.48–2.03)	0.90 (0.49–1.64)	0.93 (0.54–1.61)	0.76 (0.15–3.83)	0.73 (0.41–1.31)
Treatments received^b^									
Chemo. (no/yes)	0.82 (0.55–1.24)	0.83 (0.54–1.28)	**1.92 (1.23–2.98)**	1.46 (0.74–2.89)	1.51 (0.87–2.62)	1.15 (0.71–1.86)	1.06 (0.68–1.63)	2.04 (0.59–7.05)	1.08 (0.66–1.77)
Tamoxifen (no/yes)	1.14 (0.78–1.66)	**0.60 (0.40–0.89)**	0.84 (0.56–1.25)	0.60 (0.33–1.09)	1.05 (0.63–1.74)	1.26 (0.81–1.96)	1.38 (0.92–2.07)	0.94 (0.33–2.63)	0.82 (0.53–1.28)
Continuous CAM Use before diagnosis^b^									
Multivit.	N/A	**3.60 (2.52–5.15)**	**1.85 (1.25–2.75)**	1.53 (0.81–2.90)	1.63 (0.99–2.70)	0.93 (0.61–1.43)	1.43 (0.97–2.13)	1.04 (0.37–2.92)	1.05 (0.68–1.63)
Single vit.	**3.61 (2.53–5.16)**	N/A	**1.58 (1.04–2.38)**	**3.87 (1.77–8.45)**	0.98 (0.58–1.65)	**1.74 (1.10–2.75)**	1.06 (0.70–1.59)	0.82 (0.28–2.35)	**2.13 (1.33–3.42)**
Botanicals	**1.79 (1.21–2.66)**	**1.59 (1.05–2.40)**	N/A	0.70 (0.39–1.27)	1.13 (0.70–1.84)	1.32 (0.86–2.03)	**1.69 (1.12–2.53)**	1.29 (0.47–3.57)	**1.73 (1.13–2.65)**
Other diet. supp.	1.38 (0.75–2.52)	**3.38 (1.62–7.05)**	0.75 (0.43–1.33)	N/A	**2.86 (1.57–5.20)**	1.12 (0.62–2.04)	1.00 (0.54–1.82)	1.23 (0.32–4.65)	**3.55 (2.02–6.24)**
Mind-body	1.58 (0.96–2.61)	0.98 (0.58–1.65)	1.04 (0.64–1.69)	**2.64 (1.42–4.89)**	N/A	**1.71 (1.04–2.79)**	**7.53 (4.50–12.62)**	**5.43 (1.93–15.28)**	**2.41 (1.48–3.94)**
Special diet	0.93 (0.60–1.43)	**1.65 (1.05–2.60)**	1.31 (0.85–2.01)	1.26 (0.67–2.35)	**1.66 (1.01–2.74)**	N/A	1.24 (0.80–1.94)	1.17 (0.39–3.51)	1.55 (0.98–2.45)
CAM practitioner	1.11 (0.72–1.71)	**2.00 (1.26–3.18)**	**1.74 (1.14–2.66)**	3.94 (2.18–7.09)	**2.34 (1.42–3.85)**	1.53 (0.97–2.43)	1.54 (0.99–2.39)	2.17 (0.74–6.37)	N/A

^a^Data collected at baseline, 1996-997; ^b^Data collected at follow-up, 2002–2004.

Bold text indicates statistically significant odds ratio (*P* < 0.05).

**Table 6:** Factors associated with *starting* (vs. continuing, stopping or never using) self-care and practitioner-based CAM after diagnosis among women in the LIBCSP, multivariable analyses, OR (95% Confidence Interval).

	Multivitamins^b^	Single vitamins/minerals^b^	Botanicals^b^	Other dietary supplements^b^	Mind-body^b^	Special diets^b^	Prayer^b^	Support groups^b^	CAM practitioners^b^
Age^a^									
Continuous	1.00 (0.97–1.03)	1.01 (0.98–1.04)	0.99 (0.95–1.04)	0.99 (0.96–1.03)	**0.93 (0.89–0.97)**	1.01 (0.97–1.05)	**0.92 (0.87–0.98)**	**0.96 (0.93–0.99)**	**0.96 (0.93–0.99)**
Race^a^									
Non-Hispanic white	1 (Ref)	1 (Ref)	1 (Ref)	1 (Ref)	1 (Ref)	1 (Ref)	1 (Ref)	1 (Ref)	1 (Ref)
All Other	2.09 (0.98–4.46)	1.37 (0.60–3.13)	**3.46 (1.33–9.01)**	0.76 (0.25–2.34)	0.93 (0.31–2.79)	**3.63 (1.53–8.63)**	**3.52 (1.02–12.10)**	0.87 (0.35–2.14)	1.18 (0.48–2.87)
Annual household income^a^									
<$25k	1 (Ref)	1 (Ref)	1 (Ref)	1 (Ref)	1 (Ref)	1 (Ref)	1 (Ref)	1 (Ref)	1 (Ref)
$25k–$49,999	1.07 (0.52–2.17)	1.30 (0.60–2.80)	0.41 (0.14–1.23)	0.93 (0.34–2.55)	0.84 (0.25–2.91)	1.17 (0.44–3.09)	0.30 (0.06–1.39)	1.39 (0.47–4.13)	**0.31 (0.13–0.72)**
$50k–$89,999	0.90 (0.42–1.93)	1.49 (0.66–3.36)	0.47 (0.16–1.43)	1.23 (0.43–3.53)	0.71 (0.21–2.38)	0.74 (0.26–2.10)	0.39 (0.10–1.61)	1.89 (0.64–5.55)	**0.39 (0.17–0.90)**
≥$90k	1.02 (0.44–2.36)	2.00 (0.83–4.85)	0.70 (0.22–2.30)	1.41 (0.46–4.34)	0.33 (0.09–1.25)	0.75 (0.24–2.33)	0.29 (0.06–1.47)	1.75 (0.57–5.38)	0.59 (0.24–1.46)
Education^a^									
≤High school grad.	1 (Ref)	1 (Ref)	1 (Ref)	1 (Ref)	1 (Ref)	1 (Ref)	1 (Ref)	1 (Ref)	1 (Ref)
Some college	0.96 (0.57–1.62)	1.03 (0.61–1.72)	1.89 (0.91–3.95)	1.64 (0.86–3.10)	2.02 (0.98–4.17)	1.13 (0.59–2.17)	2.26 (0.80–6.37)	1.24 (0.66–2.31)	1.47 (0.80–2.68)
College graduate	0.82 (0.41–1.65)	0.89 (0.44–1.78)	0.82 (0.31–2.21)	0.93 (0.39–2.25)	1.76 (0.73–4.26)	0.86 (0.36–2.07)	0.87 (0.22–3.46)	**2.27 (1.13–4.56)**	1.05 (0.48–2.27)
Postgraduate	1.54 (0.84–2.82)	0.97 (0.51–1.81)	1.57 (0.66–3.72)	1.36 (0.64–2.87)	**3.90 (1.76–8.62)**	0.87 (0.38–2.00)	2.01 (0.59–6.80)	1.75 (0.90–3.40)	1.49 (0.75–2.96)
BMI at diagnosis^a^									
<25	1 (Ref)	1 (Ref)	1 (Ref)	1 (Ref)	1 (Ref)	1 (Ref)	1 (Ref)	1 (Ref)	1 (Ref)
25–29	1.01 (0.64–1.60)	0.86 (0.54–1.38)	1.02 (0.53–1.96)	1.10 (0.63–1.93)	1.27 (0.69–2.33)	0.73 (0.41–1.28)	0.38 (0.13–1.12)	1.35 (0.82–2.23)	1.07 (0.63–1.82)
≥30	1.05 (0.61–1.81)	1.06 (0.61–1.85)	1.54 (0.69–3.45)	1.28 (0.64–2.57)	**2.11 (1.03–4.33)**	**0.23 (0.08–0.64)**	1.31 (0.47–3.63)	0.89 (0.46–1.74)	1.30 (0.70–2.40)
Menopausal (meno.) status^a^									
Premeno.	1 (Ref)	1 (Ref)	1 (Ref)	1 (Ref)	1 (Ref)	1 (Ref)	1 (Ref)	1 (Ref)	1 (Ref)
Postmeno.	0.92 (0.50–1.68)	0.83 (0.45–1.56)	0.85 (0.38–1.87)	1.12 (0.54–2.34)	0.52 (0.24–1.10)	0.84 (0.38–1.84)	0.81 (0.26–2.47)	1.20 (0.65–2.23)	0.90 (0.47–1.75)
Mammogram^a^									
Never/>5 yrs ago	1 (Ref)	1 (Ref)	1 (Ref)	1 (Ref)	1 (Ref)	1 (Ref)	1 (Ref)	1 (Ref)	1 (Ref)
<5 yrs ago	1.06 (0.60–1.87)	0.91 (0.52–1.62)	1.07 (0.34–3.38)	1.53 (0.48–4.86)	1.20 (0.56–2.57)	0.86 (0.42–1.73)	1.70 (0.60–4.81)	0.97 (0.52–1.78)	1.66 (0.71–3.86)
Physical activity (h/wk)^a^									
0	1 (Ref)	1 (Ref)	1 (Ref)	1 (Ref)	1 (Ref)	1 (Ref)	1 (Ref)	1 (Ref)	1 (Ref)
0–0.69	0.70 (0.40–1.23)	1.44 (0.82–2.51)	1.03 (0.42–2.56)	1.07 (0.54–2.15)	1.39 (0.62–3.07)	1.57 (0.70–3.52)	0.47 (0.14–1.55)	0.71 (0.35–1.42)	0.89 (0.46–1.75)
0.70–2.6	0.97 (0.57–1.66)	1.16 (0.65–2.06)	1.28 (0.56–2.92)	0.57 (0.27–1.21)	1.34 (0.62–2.90)	1.99 (0.91–4.33)	0.70 (0.24–1.99)	1.22 (0.65–2.29)	0.82 (0.43–1.57)
≥2.7	0.71 (0.40–1.27)	0.85 (0.46–1.57)	1.37 (0.60–3.10)	1.06 (0.53–2.13)	1.16 (0.52–2.59)	1.81 (0.83–3.99)	0.94 (0.33–2.68)	0.90 (0.47–1.73)	1.14 (0.60–2.15)
Cigarette smoking status^a^									
Never	1 (Ref)	1 (Ref)	1 (Ref)	1 (Ref)	1 (Ref)	1 (Ref)	1 (Ref)	1 (Ref)	1 (Ref)
Current	0.69 (0.39–1.22)	0.81 (0.46–1.43)	1.47 (0.66–3.31)	0.72 (0.34–1.55)	0.92 (0.45–1.88)	0.77 (0.36–1.65)	1.04 (0.39–2.72)	0.72 (0.38–1.35)	1.23 (0.67–2.28)
Former	0.83 (0.53–1.30)	0.80 (0.51–1.26)	1.32 (0.70–2.51)	1.23 (0.72–2.09)	0.93 (0.51–1.69)	1.13 (0.63–2.00)	0.49 (0.18–1.34)	0.63 (0.38–1.05)	1.12 (0.66–1.88)
Alcohol use^a^									
Never	1 (Ref)	1 (Ref)	1 (Ref)	1 (Ref)	1 (Ref)	1 (Ref)	1 (Ref)	1 (Ref)	1 (Ref)
Ever	0.80 (0.52–1.22)	0.85 (0.55–1.30)	1.08 (0.57–2.03)	1.13 (0.66–1.95)	0.77 (0.44–1.35)	0.88 (0.50–1.55)	1.66 (0.68–4.07)	1.11 (0.68–1.81)	**0.50 (0.31–0.81)**
Fruit and vegetable intake (servings/wk)^a^									
0–34	1 (Ref)	1 (Ref)	1 (Ref)	1 (Ref)	1 (Ref)	1 (Ref)	1 (Ref)	1 (Ref)	1 (Ref)
≥35	0.70 (0.46–1.08)	0.77 (0.50–1.18)	0.92 (0.51–1.64)	1.04 (0.63–1.72)	0.89 (0.51–1.54)	0.93 (0.54–1.58)	0.56 (0.23–1.35)	1.20 (0.77–1.89)	**1.75 (1.10–2.78)**
Stage at diagnosis^a^									
In situ	1 (Ref)	1 (Ref)	1 (Ref)	1 (Ref)	1 (Ref)	1 (Ref)	1 (Ref)	1 (Ref)	1 (Ref)
Invasive	0.85 (0.47–1.54)	0.58 (0.32–1.06)	0.96 (0.43–2.18)	0.73 (0.37–1.45)	0.84 (0.38–1.89)	0.82 (0.39–1.71)	0.51 (0.15–1.68)	**2.14 (0.99–4.64)**	1.02 (0.53–1.97)
Treatments received^b^									
Chemo. (no/yes)	1.00 (0.62–1.60)	1.12 (0.69–1.82)	0.88 (0.45–1.74)	0.58 (0.31–1.07)	1.04 (0.55–1.99)	1.08 (0.59–1.99)	1.78 (0.65–4.93)	1.24 (0.74–2.08)	0.75 (0.43–1.29)
Tamoxifen (no/yes)	1.39 (0.89–2.17)	**1.86 (1.17–2.95)**	0.92 (0.50–1.68)	1.54 (0.88–2.69)	1.41 (0.79–2.52)	0.69 (0.40–1.19)	1.32 (0.57–3.06)	1.17 (0.72–1.90)	1.04 (0.64–1.70)
Continuous CAM Use before diagnosis^b^									
Multivit.	N/A	**0.51 (0.33–0.77)**	1.23 (0.66–2.30)	1.12 (0.66–1.91)	**0.57 (0.33–1.00)**	0.97 (0.56–1.67)	0.59 (0.26–1.33)	0.99 (0.61–1.60)	0.85 (0.52–1.39)
Single vit.	**0.52 (0.34–0.78)**	N/A	**9.92 (3.69–26.63)**	1.17 (0.68–2.01)	1.49 (0.82–2.73)	0.92 (0.52–1.62)	0.63 (0.27–1.49)	1.23 (0.73–2.05)	0.93 (0.56–1.53)
Botanicals	1.13 (0.72–1.79)	**1.62 (1.02–2.57)**	N/A	**2.02 (1.20–3.41)**	**1.98 (1.15–3.41)**	**2.42 (1.41–4.15)**	1.65 (0.74–3.71)	1.26 (0.79–2.00)	1.49 (0.91–2.43)
Other diet. supp.	0.93 (0.44–1.94)	0.39 (0.15–1.05)	**2.72 (1.39–5.33)**	N/A	1.19 (0.55–2.58)	1.04 (0.44–2.47)	1.55 (0.48–5.03)	1.72 (0.93–3.16)	0.88 (0.41–1.90)
Mind-body	0.68 (0.36–1.26)	0.88 (0.47–1.64)	1.36 (0.69–2.68)	1.69 (0.91–3.12)	N/A	0.58 (0.27–1.26)	0.66 (0.21–2.04)	**2.39 (1.40–4.05)**	0.80 (0.42–1.53)
Special diet	0.69 (0.41–1.18)	**0.58 (0.33–1.00)**	1.09 (0.58–2.06)	1.17 (0.66–2.07)	1.12 (0.61–2.06)	N/A	0.95 (0.37–2.48)	1.12 (0.67–1.86)	0.63 (0.35–1.13)
CAM practitioner	0.93 (0.56–1.56)	**0.55 (0.31–0.97)**	0.93 (0.49–1.75)	**0.50 (0.26–0.96)**	1.13 (0.63–2.04)	0.68 (0.35–1.33)	1.00 (0.39–2.60)	1.35 (0.82–2.23)	N/A

^a^Data collected at baseline, 1996-1997; ^b^Data collected at follow-up, 2002–2004.

Bold text indicates statistically significant odds ratio (*P* < 0.05).

## Abbreviations

CAM: Complementary and alternative medicine
LIBCSP: Long Island Breast Cancer Study Project
BMI: Body mass index.
